# Pyrrolidine Dithiocarbamate Prevents Neuroinflammation and Cognitive Dysfunction after Endotoxemia in Rats

**DOI:** 10.3389/fnagi.2016.00175

**Published:** 2016-07-21

**Authors:** Min Hui Kan, Ting Yang, Hui Qun Fu, Long Fan, Yan Wu, Niccolò Terrando, Tian-Long Wang

**Affiliations:** ^1^Department of Anesthesiology, Xuanwu Hospital, Capital Medical UniversityBeijing, China; ^2^Department of Anatomy, Capital Medical UniversityBeijing, China; ^3^Department of Medicine, Division of Nephrology, Durham VA and Duke University Medical CentersDurham, NC, USA; ^4^Department of Anesthesiology, Basic Science Division, Duke University Medical CenterDurham, NC, USA

**Keywords:** ageing, astrocytes, IL-1β, lipopolysaccharide, NF-κB, PSD-95

## Abstract

Systemic inflammation, for example as a result of infection, often contributes to long-term complications. Neuroinflammation and cognitive decline are key hallmarks of several neurological conditions, including advance age. The contribution of systemic inflammation to the central nervous system (CNS) remains not fully understood. Using a model of peripheral endotoxemia with lipopolysaccharide (LPS) we investigated the role of nuclear factor-κB (NF-κB) activity in mediating long-term neuroinflammation and cognitive dysfunction in aged rats. Herein we describe the anti-inflammatory effects of pyrrolidine dithiocarbamate (PDTC), a selective NF-κB inhibitor, in modulating systemic cytokines including tumor necrosis factor (TNF)-α and interleukin-1β (IL-1β) and CNS markers after LPS exposure in aged rats. In the hippocampus, PDTC not only reduced neuroinflammation by modulating canonical NF-κB activity but also affected IL-1β expression in astrocytes. Parallel effects were observed on behavior and postsynaptic density-95 (PSD95), a marker of synaptic function. Taken together these changes improved acute and long-term cognitive function in aged rats after LPS exposure.

## Introduction

Inflammation is a critical risk factor in the development of neurological complications; neuroinflammation in particular has become a key hallmark of several conditions including neurodegenerative diseases and psychiatric illness (Lynch, 2010; Najjar et al., 2013; Heneka et al., 2015). Systemic perturbations, like infection, are known to affect the central nervous system (CNS) causing a constellation of symptoms referred to as “sickness behavior” (Dantzer, 2004). These events may predispose to significant complications, non-resolved inflammation, and persistent cognitive impairments especially to frail and elderly subjects (Perry et al., 2007; Cunningham et al., 2009). Microglia activation is a known hallmark of neuroinflammation (Lucin and Wyss-Coray, 2009) but other cell types in the CNS like astrocytes have been also implicated in inflammatory signaling and brain plasticity (Dong and Benveniste, 2001; Capani et al., 2016). During ageing, astrocytes demonstrate phenotypical changes that have been associated with cells that express a senescence-associated secretory phenotype (SASP; Campisi, 2005). These features include enhanced expression of glial fibrillary acidic protein (GFAP), increased expression of several pro-inflammatory cytokines, and a sustained low-level oxidative stress (Cotrina and Nedergaard, 2002; Salminen et al., 2011). Within the aged brain, astrocyte activation has been implicated with higher cytokines levels including tumor necrosis factor (TNF)-α, interleukin (IL)-1β, and IL-6 (Campuzano et al., 2009). These cytokines are known to interfere with neuronal function, synaptic plasticity, and memory processes (Henry et al., 2009; Cunningham, 2013).

In our previous work we reported a specific role for IL-1β and nuclear factor-κB (NF-κB) signaling in astrocytes causing long-term neuroinflammation up to day 30 after lipopolysaccharide (LPS) exposure (Fu et al., 2014). NF-κB is a crucial regulator of immunity (Karin and Lin, 2002) and an important nuclear transcription factor involved in SASP-associated inflammation and inflammatory signaling (Salminen et al., 2012). Phosphorylation of the IKKβ pathway is central to the canonical activation of NF-κB, thus allowing nuclear translocation and transcription of several inflammatory genes. Many of these genes, including TNF-α, IL-1β, IL-6, and cyclooxygenase-2 (COX-2) mediate neuroinflammation in a number of conditions (Shih et al., 2015). The aim of the current study was to evaluate the protective effects of pyrrolidine dithiocarbamate (PDTC), a well-described inhibitor of the canonical NF-κB signaling pathway with low-molecular weight and blood-brain barrier (BBB) permeability (Schreck et al., 1992; Ziegler-Heitbrock et al., 1993; Chabicovsky et al., 2010) on neuroinflammation and memory function after LPS exposure in aged rats. Several studies have described protective effects of PDTC through suppression of NF-κB translocation into the nucleus (Ohta et al., 2002; Hirata et al., 2007), yet the full therapeutic potential of PDTC in the context of neuroinflammation and immune-to-brain signaling pathway is poorly known. Based on the above, we hypothesized that PDTC, would improve memory outcome in aged rats. Overall, we demonstrate a key role for NF-κB inhibition in preventing systemic inflammation, prolonged neuroinflammation, and behavioral changes in the hippocampus of aged rats. PDTC pretreatment aside from reducing IL-1β up regulation in astrocytes also prevented changes in postsynaptic density-95 (PSD-95), a major scaffolding protein in the excitatory PSD, thus improving neurological function.

## Materials and Methods

### Animal

Male Wistar rats (20 months old) weighing 550–850 g were used in all the experiments. Rats were bred and maintained under standardized housing conditions with controlled temperature, humidity, and 12-h light/dark cycle. Standard food chow and water were provided *ad libitum*. The experimental protocol was approved by the Capital Medical University Biomedical Ethics Committee Experimental Animal Ethics Branch (Approval No. LA2012-38) and all efforts were made to minimize pain and suffering of the animals.

Rats were randomly separated into four groups:

1. Vehicle control (0.9% NaCl i.p.),
2. LPS (2 mg/kg i.p., 055:B5, Sigma, St Louis, MO, USA),
3. LPS + PDTC (LPS + PDTC) (LPS and PDTC 30 mg/kg i.p., P8765, Sigma, St. Louis, MO, USA), and
4. PDTC alone (30 mg/kg i.p.).

LPS dosing was based on previous work (Fu et al., 2014). PDTC was administered 1 h before LPS injection at a dose (30 mg/kg) shown to inhibit NF-κB activation (Cvek and Dvorak, 2007). Vehicle group received an identical volume of saline.

### Sample Preparation

A cohort of rats was sacrificed at 0.5 h, 2 h, 6 h, 1 day, 3 days, 7 days, 15 days, and 30 days (*n* = 8 per time point per group) after LPS or saline injection. Under deep isoflurane anesthesia (Forene, Abbott Laboratories, Queensborough, UK) blood was taken from the inferior vena cava. Samples were allowed to clot for 2 h before centrifugation at 4°C for 20 min at 1000× g. Serum was collected and stored at −80°C.

Brains were rapidly dissected following decapitation. All dissections were performed on an ice-cold frosted glass plate. The entire brains (*n* = 4 per time point) were mounted in OCT compound (Sakura Finetek USA, Inc., Torrance, CA, USA), frozen in liquid nitrogen, and stored at −80°C for later immunofluorescence. The hippocampal tissues were isolated from the entire brains from the remaining batch (*n* = 4 per time point), frozen in liquid nitrogen, and stored at −80°C used for RT-PCR and ELISA analyses. The investigator was blinded to the tested groups for biochemical and behavioral analyses.

### Immunofluorescence

Brains were processed on a freezing microtome (CM1850, Leica Microsciences, Mannheim, Germany) and consecutive 20-μm thick hippocampal coronal sections were selected from Bregma −2.30 and Bregma −3.60 according to the atlas by Paxinos and Watson. Briefly, sections were post-fixed in ice-cold 4% paraformaldehyde for 15 min and rinsed in PBS four times for 10 min each time. The sections were permeabilized using 0.3% Triton X-100 (Sigma-Aldrich, St Louis, MO, USA) for 1 h, blocked with 5% horse serum (8178102, Gibco) for 1 h at room temperature and then incubated with primary antibodies: goat anti-IL-1β IgG (1:100; catalog number AF-501-NA, R&D, Systems Inc., Minneapolis, MN, USA), rabbit anti-PSD-95 (1:100; catalog number 04-1066, Millipore, Billerica, MA, USA), rabbit anti-GFAP IgG (1:1000; catalog number Z0334; Dako), rabbit anti-IBA-1 IgG (1:500, catalog number 019-19741 Wako) and mouse anti-NeuN IgG (1:100; catalog number MAB360, Millipore) for 2 h at room temperature, and then overnight at 4°C. The sections were washed three times with PBS and incubated for 2 h with secondary antibodies: Alexa-594-coupled donkey anti-goat IgG (1:500; catalog number: A11058, Invitrogen), Alexa-594-coupled donkey anti-rabbit IgG (1:500; catalog number: A21207, Invitrogen), Alexa 488-coupled donkey anti-rabbit IgG (1:500; catalog number: A21206, Invitrogen), Alexa-488-coupled donkey anti-mouse IgG (1:500; catalog number: A21202, Invitrogen, Paisley, UK). Negative control sections in which primary antibodies or secondary antibodies were replaced by PBS showed no labeled cells. The nuclei of tissues were counterstained with Hoechst 33342 (1:1000; Roche, Mannheim, Germany). Sections from all time points were stained simultaneously to provide uniform conditions for subsequent quantitative analysis by fluorescence staining. Samples were analyzed with a confocal microscope (Leica TCS SP5, Leica, Benshein, Germany) and the rate of IL-1β positive expression in the GFAP-positive astrocytes of the hippocampal DG region were analyzed using Adobe Photoshop CS3V10.0.1.0. Mean gray values of PSD-95 were measured using an image analyzing software (Optimas 6.5, CyberMetrics, Scottsdale, AZ, USA). Ten visual fields were counted for each section, and 10 values of positive expression were counted and the mean value calculated by averaging the counts from the results of the three experiments at different time points (Fu et al., 2014).

### mRNA and RT-PCR

Total RNA was extracted from the hippocampus using Trigol (NEP019-2, Dingguo Changsheng) and DNA contaminants removed by RQ1 RNase-free DNase (M610A, Promega). The integrity of total RNA was measured by agarose gel electrophoresis and cDNA was synthesized with Toyobo Reverse transcription Kit (including M-MLV and 5× RT Buffer) according to the manufacturer’s protocol (TRT-101, Toyobo). DNA amplification was carried out using Sybr Green I (10×) (GG1301-500UL, Genviw) for 35 cycles (each cycle consisting of denaturation for 30 s at 94°C, annealing for 30 s at 56°C or 47°C, extension for 30 s at 72°C). Primers used are described in Table 1. The fold expression in gene was determined by double-standard curves method of relative quantification PCR. Data are expressed as the relative level of the target gene in the hippocampus normalized to the endogenous control (GAPDH) and relative to the control group.

**Table 1:** **Sequences of primers for RT-PCR**.

	Forward primers (5′ → 3′)	Reverse primers (5′ → 3′)
IL-1β	ATGAGAGCATCCAGCTTCAAATC	CACACTAGCAGGTCGTCATCATC
NF-κB	TCCATCAAATCTTCCCG	CCTTTCCTTGTCCAGCA
I-κBα	ACCCCTCTCCATCTTGCC	CATCAGCCCCACACTTCA
PSD-95	ACAACCAAGAAATACCGC	ATACTCCATCTCCCCCTC
GAPDH	ACAGCAACAGGGTGGTGGAC	TTTGAGGGTGCAGCGAACTT

### Enzyme-Linked Immunosorbent Assay

Isolated hippocampal tissues were homogenized in 100 mg tissue/ml RIPA Lysis Buffer (20-188, Millipore, MA, USA). Immediately prior to use, protease inhibitor (Roche Diagnostics, Indianapolis, IN, USA) and phosphatase inhibitor (pepstatin, Roche Diagnostics, Indianapolis, IN, USA) were added into the RIPA Lysis Buffer. The resulting suspension was sonicated with an ultrasonic cell disrupter (Bandelin, OSTC) and centrifuged at 14,000× g at 4°C for 15 min. Supernatant was collected and stored at −80°C. Nuclear protein extraction was performed with NE-PER^TM^ Nuclear and Cytoplasmic Extraction Reagents (78833, Thermo) according to the manufacturer’s instructions, samples were stored at −80°C. Protein quantification was performed using BCA Protein Assay Kit (23227, Thermo, USA). ELISAs were performed according to the manufacturer’s instructions (IL-1β RLB00; TNF-α RTA00, R&D Systems; NF-κB ab133128; p-IκBα ab176643, Abcam; PSD-95, LS-F6865, LifeSpan BioSciences, Inc.) and optical density determined at 450 with a microplate reader (BioRad, Richmond, CA, USA).

### Morris Water Maze Test

One day after treatment, learning ability and memory for spatial orientation of the rats were assessed by Morris water maze (MWM) test in a separate cohort of animals (*n* = 8–10/group) as previously described with some modification (Yang et al., 2012). The MWM consisted of a circular black painted pool and was placed in a dimly lit room with several visual clues around. The pool was filled to a depth of 20 cm water (25 ± 2°C) and with a hidden submerged platform located 1.5 cm below the water surface in one fixed quadrant. Rats were gently placed in one quadrant facing the wall of MWM pool. The rats were allowed to swim for 60 s to locate the hidden platform during each trial. When successful, the rat was allowed to stay for 5 s on the platform. If unsuccessful within 60 s, the rat was then physically placed on the platform for 20 s. A 3–5 min interval was allowed between each trial. The time spent to locate the platform, swimming speed and distance were recorded by a video camera and analyzed by Videomot software (version 2.4.50923; TSE Systems, Bad Homburg, Germany). Four spatial acquisition trials were performed on each rat per day with the starting location in each quadrant and five consecutive days of training were conducted (day 1–5). On day 7, a probe trial was conducted with the platform removed. Rats were placed in the quadrant opposite to the original location of the platform, and allowed to swim in the pool for 30 s. The times crossover the platform location and the percentage of time spent in the previous platform quadrant were recorded. From day 31 to 35, spatial acquisition trials were repeated followed with a probe trial on day 37. The time line of MWM protocol is shown in Figure 5A.

### Statistical Analyses

For statistical analyses Prism v5 software (GraphPad Software Inc., La Jolla, CA, USA) was used. All values in the figures are presented as mean ± SEM. MWM test was analyzed by repeated-measures two-way ANOVA followed by Bonferroni *post hoc* analysis. All other data were analyzed by two-way ANOVA followed by Bonferroni *post hoc* analysis. *P* values < 0.05 were considered statistically significant.

## Results

### PDTC Reduces Pro-Inflammatory Systemic Cytokines After LPS Administration

**Figure 1:**
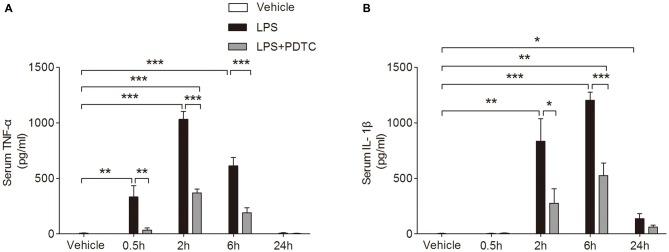
**Effects of pyrrolidine dithiocarbamate (PDTC) on lipopolysaccharide (LPS)-induced tumor necrosis factor (TNF)-α and interleukin (IL)-1β in serum.** Treatment with PDTC significantly reduced serum levels of TNF-α at 0.5 h, 2 h, and 6 h compared to LPS group **(A)**. Levels of IL-1β were also reduced at 2 h and 6 h, returning to baseline after 24 h **(B)**. Data are expressed as mean ± SEM (*n* = 4) and compared by 2-way ANOVA with Bonferroni *post hoc* analysis. *p < 0.05, **p < 0.01, ***p < 0.001.

The circulating TNF-α and IL-1β levels were significantly affected by LPS and PDTC (*F*_(2,34)_ = 152.8 and 40.92, *p* < 0.0001 respectively). *Post hoc* comparisons of two-way ANOVA showed serum TNF-α significantly increased at 0.5 h, 2 h and 6 h after LPS injection compared to the vehicle group (333.10 ± 102.90, 1033.00 ± 68.55 and 614.20 ± 75.89 vs. 6.28 ± 1.50 pg/ml, *p* < 0.01 and *p* < 0.001 respectively, Figure 1A); while serum IL-1β was significantly increased at 2 h, 6 h and 24 h after LPS injection compared to the vehicle group (835.40 ± 203.00, 1203.00 ± 74.31 and 136.80 ± 44.46 vs. 3.59 ± 1.53 pg/ml, *p* < 0.01, 0.001 and 0.05 respectively, Figure 1B). PDTC pretreatment significantly decreased serum TNF-α from 0.5 h to 6 h compared to LPS group (*p* < 0.01 and *p* < 0.001 respectively, Figure 1A). In addition, the LPS induced increase of circulating IL-1β was significantly reduced by PDTC pretreatment at 2 h and 6 h (*p* < 0.05, *p* < 0.001 respectively, Figure 1B).

### Effects of PDTC on Astrocytes and IL-1β Expression in the Hippocampus

**Figure 2:**
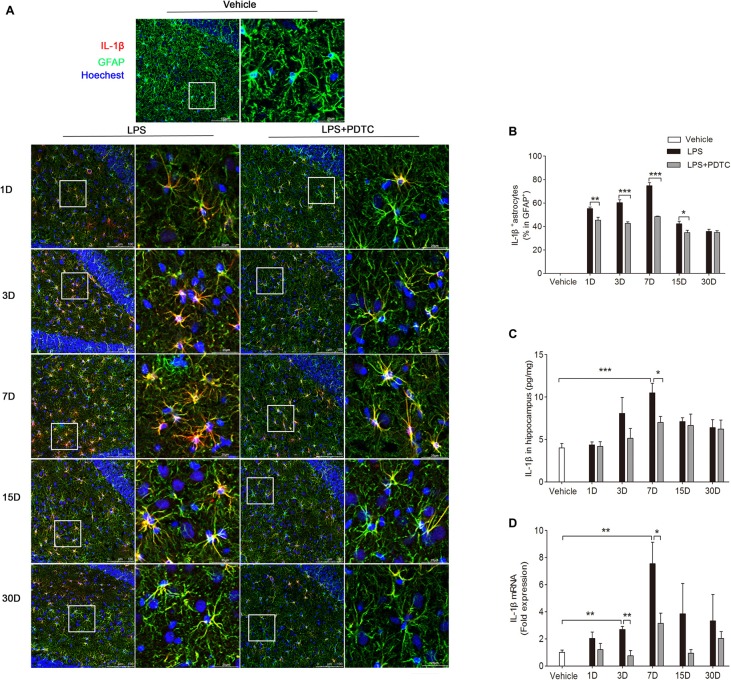
**IL-1β expression in astrocytes after LPS is reduced by PDTC.** Confocal immunofluorescence images of IL-1β/glial fibrillary acidic protein (GFAP) in the hippocampus DG region **(A)**. The ratio of IL-1β-positive astrocytes was significantly reduced in LPS + PDTC group compared to LPS **(B)**. IL-1β protein levels from hippocampal homogenates was affected by PDTC treatment especially on day 7 **(C)**. mRNA expression of IL-1β was also attenuated at all time points, in particular day 3 and 7 **(D)**. Data are expressed as mean ± SEM (*n* = 4) and compared by 2-way ANOVA with Bonferroni *post hoc* analysis. *p < 0.05, **p < 0.01, ***p < 0.001. Scale bars = 100 μm; insets = 25 μm.

Previous studies have shown that acute systemic inflammation triggered by a single administration of LPS resulted in chronic neuroinflammation (Qin et al., 2007; Gao et al., 2011). Here we focused on the effects of PDTC on GFAP-positive astrocytes in hippocampus DG region as minimal immunoreactivity was observed in microglia by Iba-1 immunofluorescence (Supplementary Figure S3). The immunofluorescence staining showed IL-1β in astrocytes was significantly affected by LPS and PDTC (*F*_(2,45)_ = 747.60, *p* < 0.0001 respectively). The IL-1β positive astrocytes in hippocampus were significantly increased from day 1 to 15 in both LPS and LPS + PDTC groups compared to the vehicle (Figures 2A,B). However, PDTC pretreatment significantly reduced LPS induced IL-1β expression in astrocytes (*p* < 0.01, *p* < 0.001, *p* < 0.001 and *p* < 0.05 vs. LPS group respectively). In addition, we measured IL-1β protein level in hippocampus using ELISA. LPS and PDTC showed significant effects on hippocampal IL-1β protein level (*F*_(1,30)_ = 34.16; *p* < 0.0001). LPS significantly increased IL-1β level in hippocampus at day 7 compared to vehicle (10.50 ± 1.12 vs. 4.02 ± 0.51 pg/mg, *p* < 0.001), which was significantly attenuated by PDTC pretreatment (7.02 ± 0.70, *p* < 0.05 vs. LPS group, Figure 2C). In addition, LPS and PDTC significantly affected the hippocampal IL-1β mRNA expression (*F*_(2,45)_ = 13.44; *p* < 0.0001). LPS i.p. caused significant increase of IL-1β mRNA expression in hippocampus at day 3 and 7 compared to vehicle (*p* < 0.01 respectively), which were significantly attenuated by PDTC pretreatment (*p* < 0.01 and *p* < 0.05 vs. LPS group respectively, Figure 2D).

### PDTC Diminished LPS Induced NF-κB p65 Activity in the Hippocampus

**Figure 3:**
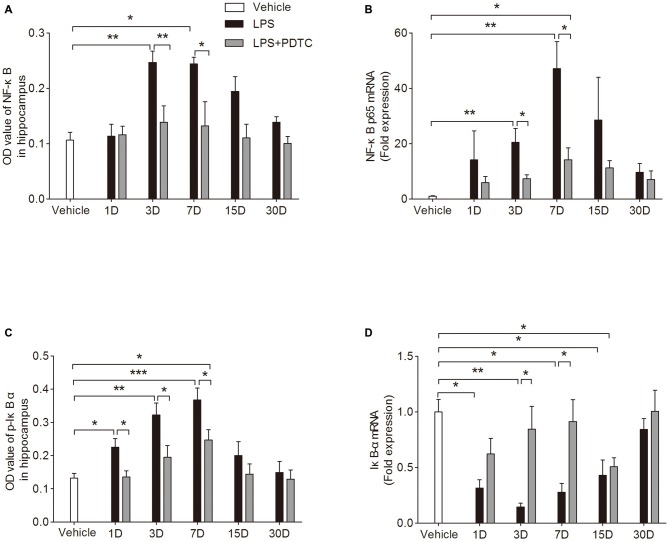
**PDTC reduced LPS-induced nuclear factor-κB (NF-κB) p65 activity in the hippocampus.** NF-κB p65 protein level and mRNA expression in hippocampus were significantly increased on day 3 and 7 after LPS exposure, which was attenuated by pre-treatment of PDTC **(A,B)**. IκB-α phosphorylation was significantly increased at day 1, 3 and 7 in LPS group compared to vehicle and LPS + PDTC groups **(C)**. LPS induced reduction of IκB-α mRNA expression in hippocampus up to 15 days, pre-treatment with PDTC reversed this effect **(D)**. Data are expressed as mean ± SEM (*n* = 4) and compared by 2-way ANOVA with Bonferroni *post hoc* analysis. *p < 0.05, **p < 0.01, ***p < 0.001.

To better understand the mechanisms underlying this neuroinflammatory response we measured NF-κB p65 and the phosphorylation of IκB-α in the hippocampus. NF-κB p65 protein and mRNA expression in hippocampus were significantly affected by LPS and PDTC treatment (*F*_(2,45)_ = 21.23 and 19.47, *p* < 0.0001 respectively). On day 3 and 7 after LPS administration NF-κB p65 protein levels and mRNA expression were significantly increased compared to vehicle (Protein level: *p* < 0.01 and *p* < 0.05 respectively; mRNA: *p* < 0.01 respectively). These changes were significantly attenuated in the LPS + PDTC group compared to LPS group (Protein level: *p* < 0.01 and *p* < 0.05 respectively, mRNA: *p* < 0.05 respectively Figures 3A,B). Further, the hippocampal IκB-α protein and mRNA expression were also affected by different treatments (*F*_(2,45)_ = 25 and 27.40, *p* < 0.0001 respectively). From day 1 to 7 after LPS administration, phosphorylated IκB-α was markedly increased in hippocampus compared to vehicle (*p* < 0.05, *p* < 0.01 and *p* < 0.001 respectively), these increase were significantly attenuated by PDTC pre-treatment (*p* < 0.05 respectively, Figure 3C). Accordingly, the mRNA expression of total IκB-α in hippocampus were significantly reduced from day 1 to 15 after LPS injection compared to vehicle group (*p* < 0.05 and *p* < 0.01 respectively). However, compared to LPS injection alone, rats with PDTC pretreatment showed reserved total IκB-α mRNA expression in the hippocampus, especially on day 3 and 7 (*p* < 0.05 respectively compared to LPS group, Figure 3D). No effects of PDTC alone were observed (Supplementary Figure S4).

### PSD-95 Expression in the Hippocampus After PDTC Treatment

**Figure 4:**
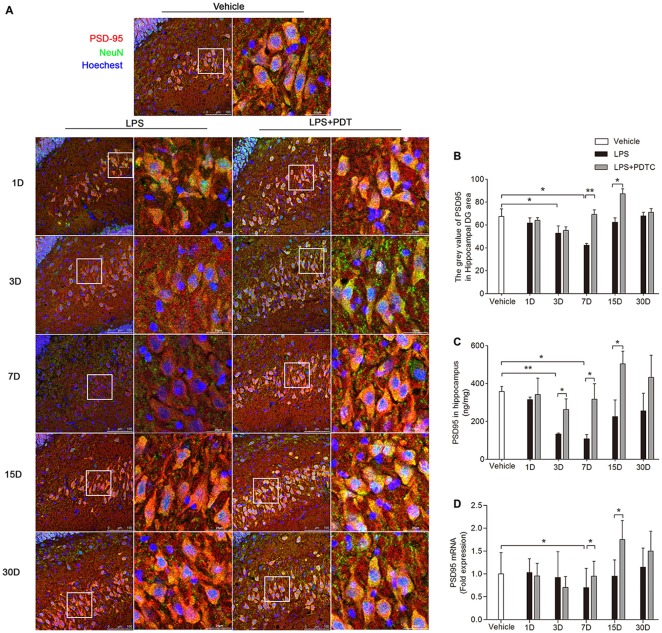
**Postsynaptic density-95 (PSD-95) expression in the hippocampus after LPS and PDTC treatment.** PSD-95 immunoreactivity was analyzed in the hippocampus, DG area **(A)**. Expression of PSD-95 in neurons was reduced on day 3 and 7, returning to baseline thereafter **(B)**. PSD-95 protein expression in the hippocampus was measured by ELISA** (C)** and RT-PCR **(D)**. There was significant increase in PSD-95 expression after PDTC treatment on day 7 and day 15 compared with the LPS group. Data are expressed as mean ± SEM (*n* = 4) and compared by 2-way ANOVA with Bonferroni *post hoc* analysis. *p < 0.05, **p < 0.01. Scale bars = 100 μm; insets = 25 μm.

Next we measured the effects of LPS administration on scaffolding proteins focusing on the major molecule, PSD-95. The immunofluorescence staining showed LPS and PDTC significantly affected the PSD-95 level in hippocampus (*F*_(2,44)_ = 9.07; *p* = 0.0005, Figures 4A,B). On both day 3 and day 7 PSD-95 was significantly decreased compared to controls (gray value as 52.86 ± 6.27 and 42.38 ± 1.59 vs. 67.42 ± 6.64, *p* < 0.05 respectively, Figure 4B). This effect was abolished by PDTC pretreatment. The protein and mRNA level of PSD-95 in hippocampus was then measured. Hippocampal PSD-95 protein level was significantly affected by different treatment (*F*_(2,45)_ = 10.99, *p* = 0.0001, Figure 4C), that LPS significantly reduced hippocampal PSD-95 on day 3 and 7 (*p* < 0.01 and *p* < 0.05 respectively), and PDTC pretreatment restored the PSD-95 level at these time points (*p* < 0.05 vs. LPS respectively). Similar changes were observed in PSD-95 mRNA expression at day 7 (Figure 4D).

### PDTC Improves LPS-Induced Cognitive Dysfunction

**Figure 5:**
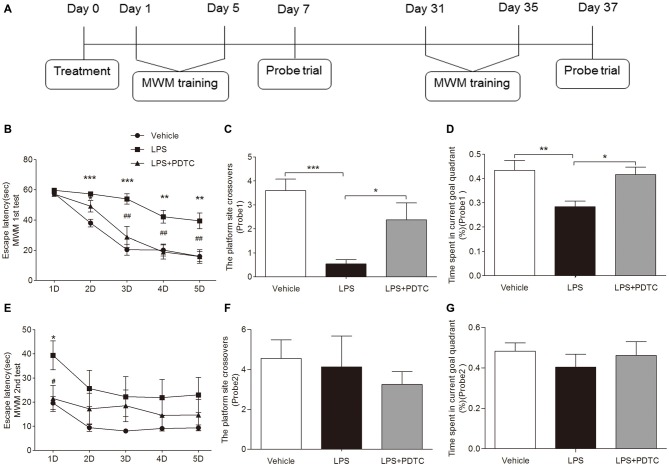
**LPS induced cognitive deficiency was ameliorated by PDTC.** The schematic figure of the water maze protocol is shown in **(A)**. Rat treated with LPS alone had prolonged escape latency from day 2 to 5 in the first round of spatial acquisition trials compared to vehicle group. This was significantly improved by PDTC **(B).** Compared to the vehicle group, rat treated with LPS also showed significant reduced platform site crossovers and time spent in the target quadrant in the probe trial on day 7, which was reversed by PDTC pre-treatment **(C,D)**. In the second round of acquisition trials, LPS only caused significant prolonged escape latency on first day (day 31) **(E)**. No differences of platform site crossovers and time spent in the target quadrant were observed between groups in the second round of probe trial **(F,G)**. Data are expressed as mean ± SEM (*n* = 8–10) and were compared by repeated measure 2-way ANOVA with Bonferroni *post hoc* analysis. *p < 0.05, **p < 0.01, ***p < 0.001 as indicated or vs. vehicle; ^#^*p* < 0.05, ^##^*p* < 0.01 vs. LPS group.

We used MWM to test learning and memory function in this model of endotoxemia. The escape latency was significantly affected by the testing days and treatment. All groups showed marked improvements in escape latencies over the 5 days of training (*F*_(3, 144)_ = 2.87, *p* < 0.0001, repeated measures ANOVA), indicating a memory in locating the escape platform. Repeated measures ANOVA revealed no interaction between training days and groups (*F*_(12, 144)_ = 1.21, *p* > 0.05). This suggests that all the rats effectively learned the task. *Post hoc* comparisons indicated that LPS significantly increased the escape latency at day 2–5 in the spatial acquisition trials compared to vehicle (*p* < 0.001 and 0.01 respectively), PDTC significantly improved the LPS-induced prolonged escape latency in the rats at day 3–5 (*p* < 0.01 respectively, Figure 5B). Furthermore, repeated-measures two-way ANOVA in the probe trial on day 7 revealed the effects of drug (*F*_(2,62)_ = 4.76, *p* < 0.05) and time (*F*_(1,62)_ = 6.56, *p* < 0.05) on the platform crossovers. *Post hoc* analysis indicated that rats with LPS challenge had significant less platform crossovers compared to vehicle or LPS + PDTC treated rats (*p* < 0.001 and *p* < 0.05 respectively). Similarly ANOVA revealed the effects of drug (*F*_(2,31)_ = 4.63, *P* < 0.05) and time (*F*_(1,31)_ = 4.98, *p* < 0.05) on the time in the target quadrant. *Post hoc* analysis indicated that rats with LPS challenge had significant less time spent in the target quadrant compared to vehicle or LPS + PDTC treated rats (*p* < 0.01 and *p* < 0.05 respectively, Figures 5C,D). However, in the second round of spatial acquisition test from day 31 to 35, rats accepted LPS alone only showed significant longer escape latency on day 31 compared to rats accepted vehicle or LPS + PDTC (39.40 ± 5.96 vs. 19.64 ± 2.65 and 21.53 ± 5.33 s, *p* < 0.05 respectively, Figure 5E). No significant differences were observed between groups regarding the platform crossover and time spent in the quadrant in the probe trial on day 37 (Figures 5F,G). The swimming speeds were similar between all groups throughout the MWM test (not shown).

## Discussion

In this study we focused on the effects of a single systemic dose of LPS in aged rats and assessed long-term changes in systemic cytokines, neuroinflammation, and behavior. Using a selective NF-κB inhibitor, PDTC, we report neuroprotective effects through inhibition of the canonical NF-κB signaling pathway, reduction of IL-1β expression in astrocytes, and stabilization of PSD-95. Taken together, PDTC offered sufficient protection to improve memory dysfunction after endotoxemia in aged rats.

Inflammation is a critical component of almost every clinical condition ranging from cancer to neurodegenerative disorders; yet non-resolving inflammation remains one of the major challenges in biomedical research (Nathan and Ding, 2010). Systemic infections can lead to significant consequences in elderly subjects and may contribute to the acceleration of pathologies like Alzheimer’s disease (Cunningham et al., 2009). LPS, a key component of Gram-negative bacteria outer membrane, has been used as a robust activator of innate immunity. Its effects on behavior have been well documented (Dantzer et al., 1998), but the mechanisms underlying cognitive impairments after endotoxemia are not fully understood. Inflammatory molecules can access the CNS through different immune-to-brain mechanisms, both blood-borne and neuronal (Capuron and Miller, 2011; Xu et al., 2014). After LPS exposure, we found a significant increase in systemic pro-inflammatory cytokines, including TNF-α and IL-1β, which initiate a pro-inflammatory response that affects long-term CNS function. LPS acts mainly peripherally, with minimal penetration of the intact BBB (Banks and Robinson, 2010). However, evidence from high-resolution magnetic resonance imaging analysis of the BBB in the living human brains found selective BBB breakdown during ageing, which may contribute to cognitive impairment (Montagne et al., 2015). Thus, it is possible that age-induced BBB opening as well as direct transport of blood-borne factors and cytokines contribute to subsequent neuroinflammation and memory dysfunction in our model (Banks et al., 1995; He et al., 2012).

To support a role for systemic inflammation in mediating secondary CNS effects, in this study treatment with PDTC was able to significantly reduce pro-inflammatory cytokines with levels returning to baseline within 24 h. TNF-α and IL-1β are two prototypical cytokines hardwired to NF-κB signaling (Lawrence, 2009) and are implicated in the development of age-related postoperative complications including delirium and postoperative cognitive dysfunction (Androsova et al., 2015). In mice, modulation of IL-1β signaling after endotoxemia using IL-1 receptor antagonist or genetic intervention (IL-1R^−/−^) prevents cognitive dysfunction by attenuating microglia activation (Terrando et al., 2010). Microglia and astrocytes play pivotal roles in surveilling the CNS microenvironment, supporting neuronal homeostasis and protecting the brain from danger signals (Streit et al., 2004). Modulation of microglia activation was also reported after PDTC treatment in LPS-induced sepsis, improving long-term behavioral outcomes (Anderson et al., 2015). Previously we described an important role for astrocytes activation and cytokine expression in this model of LPS-induced neuroinflammation (Fu et al., 2014). Here we found PDTC was able to attenuate IL-1β expression in GFAP-positive cells in the hippocampus, confirming an important role for IL-1β and NF-κB signaling in ensuing CNS complications. In addition, pretreatment with PDTC regulated NF-κB activity by inhibiting IκB-α phosphorylation and p65 translocation into the nucleus. IκB phosphorylation is critical for activating the canonical and alternative NF-κB pathways and allowing nuclear translocation and downstream gene activation (Karin and Lin, 2002). Given PDTC is well tolerated in models of acute and chronic inflammation (Cuzzocrea et al., 2002), this could represent a suitable target for novel therapeutic discoveries.

Aside the effects of PDTC on NF-κB signaling we also describe a novel interaction with the excitatory postsynaptic marker, PSD-95. Synaptic proteins, especially PSD-95, play a critical role in regulating dendritic spine morphogenesis and plasticity, which are closely implicated in memory function and cognition (El-Husseini et al., 2000). Loss of PSD-95 coincided with NF-κB activation in the hippocampus on day 3 and 7 after LPS exposure, suggesting neuroinflammation impairs synaptic plasticity by modulating scaffolding protein at glutamatergic excitatory synapses. PSDs are essential structures for N-methyl-D-aspartate receptor signaling, thus exerting critical functions in synaptic processes, glutamate excitatory neurotransmission and calcium influx (Kennedy, 2000). Inflammation can impair synaptic plasticity especially during the ageing process or as a result of injury (Barrientos et al., 2006; Terrando et al., 2013). Changes in long-term potentiation, the molecular surrogate for memory dysfunction, have been implicated in LPS-induced changes in memory and behavior (Vereker et al., 2000). In our study PDTC rescued LPS-induced memory impairment as a result of its anti-inflammatory effects on NF-κB/cytokines signaling and stabilization of PSD-95 in the hippocampus. Improved memory function after PDTC treatment has been reported in other models of neuroinflammation and cognitive disorders (Li et al., 2012; Zhang et al., 2014). Since PDTC compounds have been used already in the clinic (Reisinger et al., 1990), inhibition of NF-κB pathway may offer therapeutic benefit for neuroinflammation and cognitive dysfunction.

Other mechanisms are likely to be involved in PDTC-mediated neuroprotection. The effects on oxidative stress are well known (Tsai et al., 1996) and these processes are tightly related with the ageing process. Increased reactive oxygen species and lipid peroxidation is found during brain ageing, contributing to microglia priming, SASP and cognitive decline (Norden and Godbout, 2013). This inherited vulnerability contributes to the prolonged and amplified immune response as noted in this study after a single LPS challenge. This “immune-priming” is likely to contribute to non-resolving inflammation and long-lasting modifications in behavior and CNS molecular markers (Bossù et al., 2012). Mechanistically, since PDTC crosses the BBB (Schreck et al., 1992; Ziegler-Heitbrock et al., 1993; Chabicovsky et al., 2010) and aged rats may have deficits in endothelial function, the drug could exert direct CNS effects both as anti-inflammatory on glia cells but also neurons. Some limitations of our study must be pointed out. We focused our study in aged rats as a clinically relevant model for a more vulnerable system to general perturbations like infection. We did not include or compare the effects of LPS and PDTC in a younger cohort, however previous studies demonstrated similar protective effects on neuroinflammation and cognitive outcomes in younger rodent models (Anderson et al., 2015). Also, the role of NF-κB modulation on different CNS cell types awaits future investigations with the use of more selective strategies to target exaggerated neuroinflammation, astrocyte activation, and brain dysfunction.

In conclusion, LPS-induced acute systemic cytokines can induce prolonged neuroinflammation in the ageing brain. Inhibition of NF-κB signaling regulates key processes in the CNS, including IL-1β up regulation in astrocytes and PSD-95 expression in the hippocampus. The anti-inflammatory and neuroprotective effects provide an important target for dampening neuroinflammation and improving cognitive decline.

## Author Contributions

Conceived and designed the experiments: MHK, T-LW, YW, HQF, LF. Performed the experiments: MHK, YW, HQF, LF. Data analyses and interpretation: MHK, TY, NT. Wrote the article: MHK, TY, NT.

## Funding

This work was supported by grants from Beijing 215 High Level Healthcare Talent Plan-Academic Leader (008-0027) and Beijing Municipal Administration of Hospitals’ ascent Plan (DFL20150802).

## Conflict of Interest Statement

The authors declare that the research was conducted in the absence of any commercial or financial relationships that could be construed as a potential conflict of interest.
