# Supplementary material for: Pyrrolidine Dithiocarbamate Prevents Neuroinflammation and Cognitive Dysfunction after Endotoxemia in Rats
Source: Front Aging Neurosci. 2016 Jul 21;8:175. doi: 10.3389/fnagi.2016.00175 (PMC4954850; doi:10.3389/fnagi.2016.00175)
Supplement: Supplementary file 1 [file Presentation_1.pdf]

Pyrrolidine Dithiocarbamate prevents Neuroinflammation and Cognitive Dysfunction after Endotoxemia in Rats

Min Hui Kan[1,2], Ting Yang[3], Hui Qun Fu[1], Long Fan[1], Yan Wu[2], Niccolò Terrando[4], Tian-Long Wang[1]*

* Correspondence:

Tian-Long Wang M.D.
E-mail: w_tl5595@hotmail.com

Supplementary figure legends

Figure 1. IL-1β expression in astrocytes.

A-D: IL-1β and GFAP individual channel representative confocal immunofluorescence images in hippocampus DG region. Scale bars =100 μm. Insets scale bar = 25 μm. **E:** Proportion of IL-1β positive astrocytes in GFAP positive astrocytes in DG region. No differences were observed between control and PDTC group. Data are expressed as mean±SEM and compared by by 2-way ANOVA with Bonferroni post hoc analysis, n=4/group.

Figure 2. PSD-95 expression in the hippocampus after LPS and PDTC treatment.

A-D: PSD-95 and NeuN individual channel representative confocal immunofluorescence images in the hippocampus DG region. Scale bars =100 μm. Insets scale bar = 25 μm. **E:** PSD-95 expression was quantified as grey value. Data are expressed as mean±SEM and compared by 2-way ANOVA with Bonferroni post hoc analysis, n=4/group.

Figure 3. IL-1β expression in microglia.

A-D: Confocal immunofluorescence images showed double labeling for IL-1β and IBA-1 in the hippocampus DG region. No evident co-localization of IL-1β and IBA-1 was observed in all groups. Scale bars =100 μm. Insets scale bar = 25 μm, n=4/group.

Figure 4. Effects of PDTC alone on neuroinflammatory markers.

Rats were treated with PDTC only and protein levels and mRNA expression of NF-κB p65 (**A, B**), p-IκB-α (**C, D**), PSD-95 (**E, F**), and IL-1β (**G, H**) were measured at different time points in the hippocampus. No significant differences were observed between PDTC and control group. Data are expressed as mean±SEM and compared by 2-way ANOVA with Bonferroni post hoc analysis, n=4/group.
