# Supplementary figures and images for: Pyrrolidine Dithiocarbamate Prevents Neuroinflammation and Cognitive Dysfunction after Endotoxemia in Rats
Source: Front Aging Neurosci. 2016 Jul 21;8:175. doi: 10.3389/fnagi.2016.00175 (PMC4954850; doi:10.3389/fnagi.2016.00175)

Fig.1

A

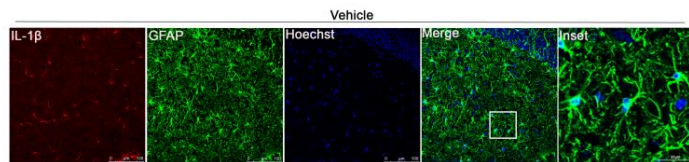

B

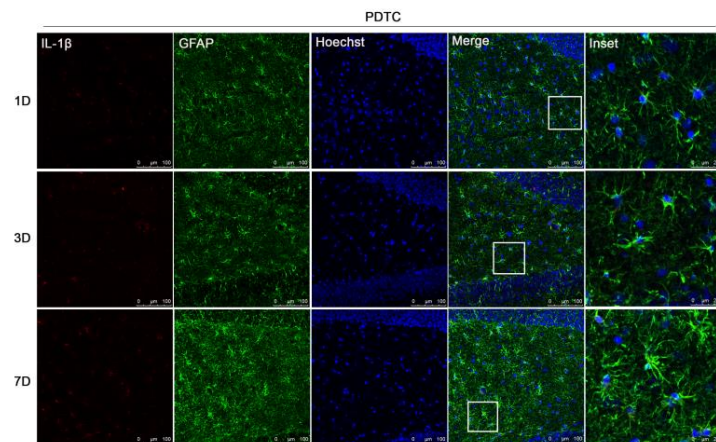

C

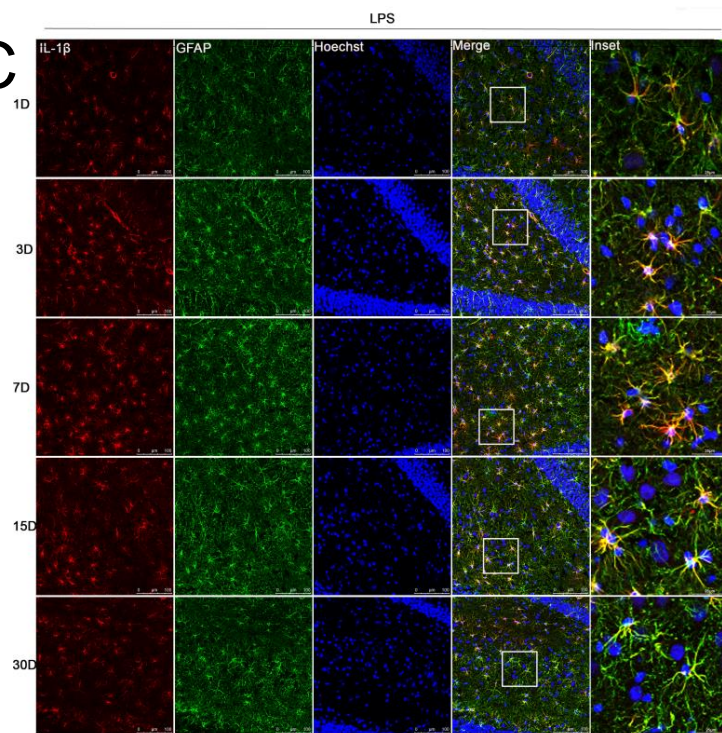

D

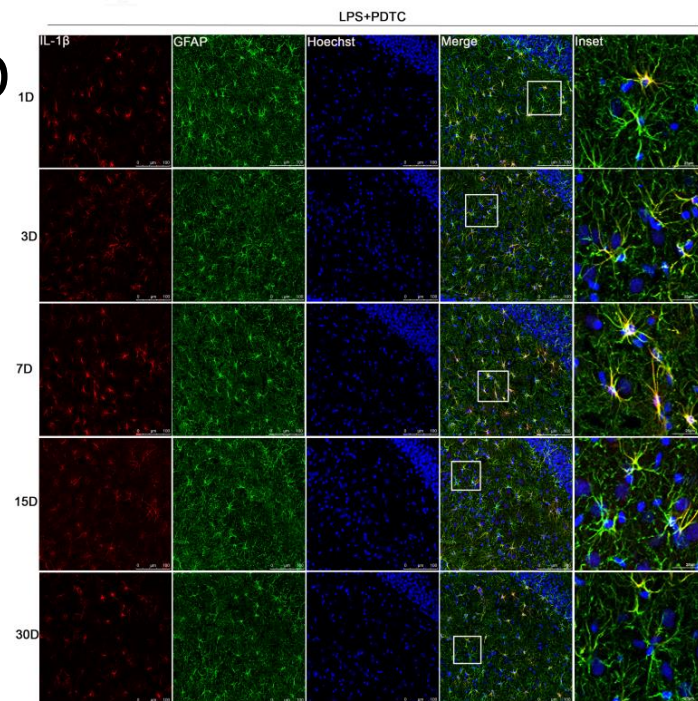

E

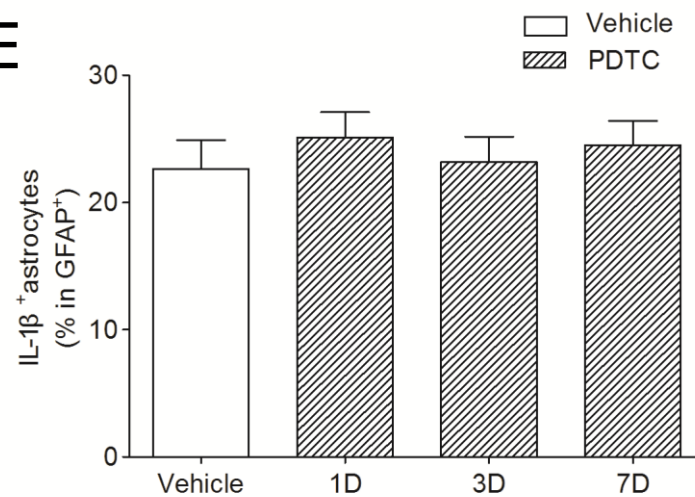

# Fig.2

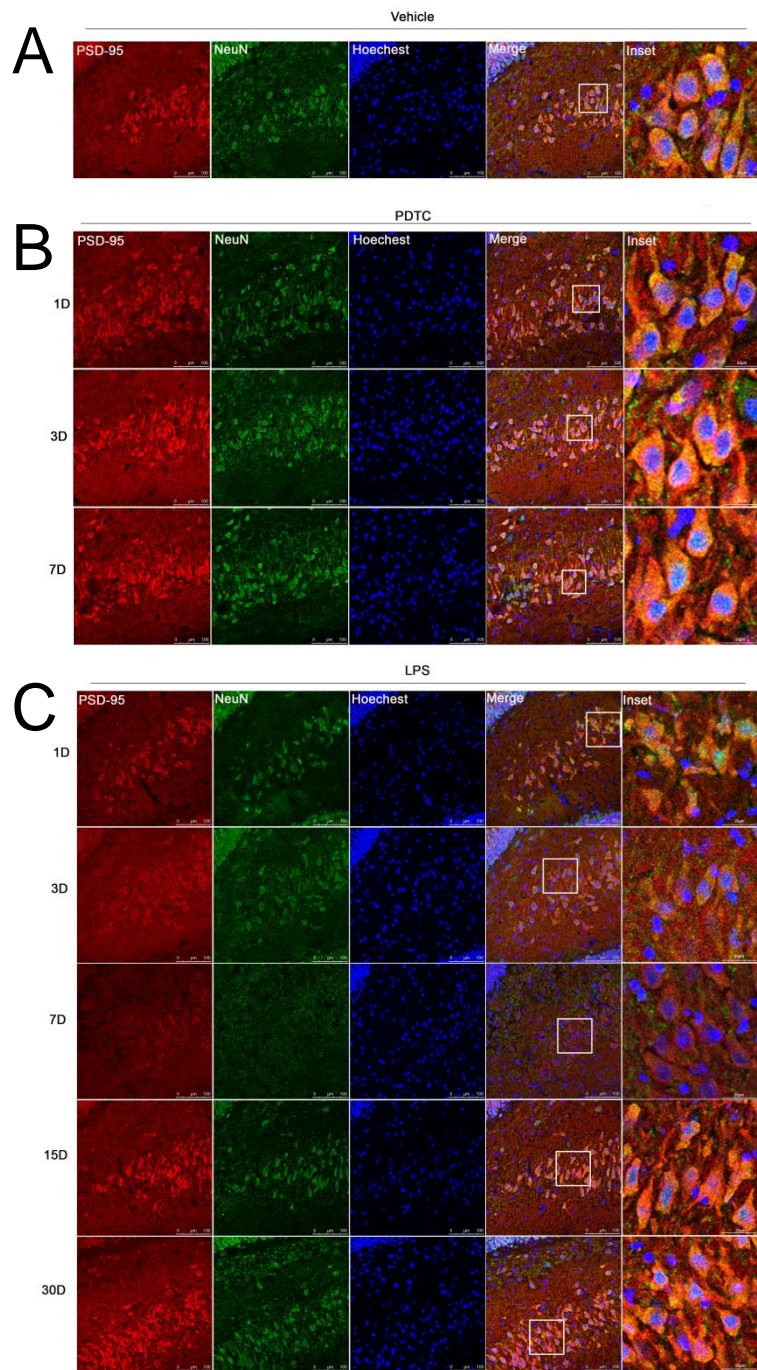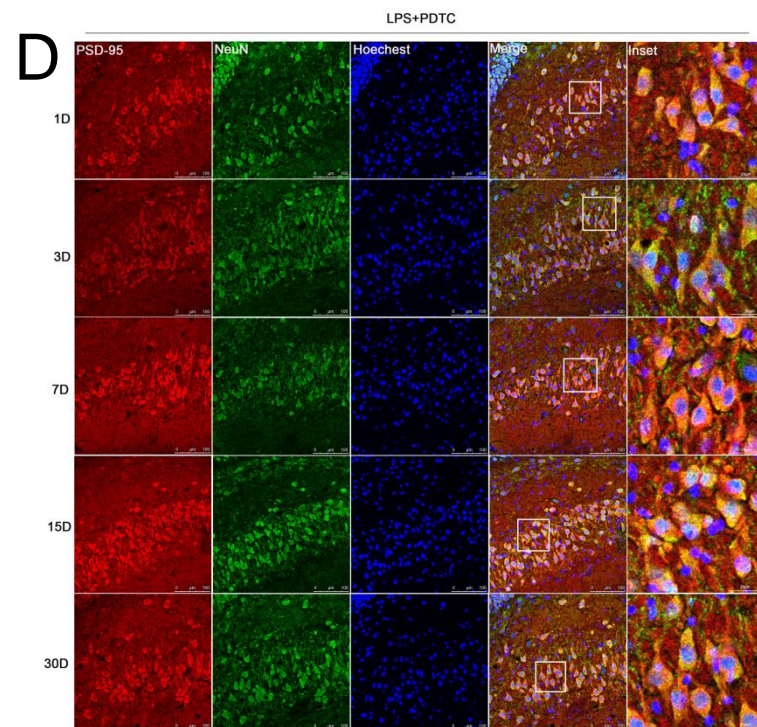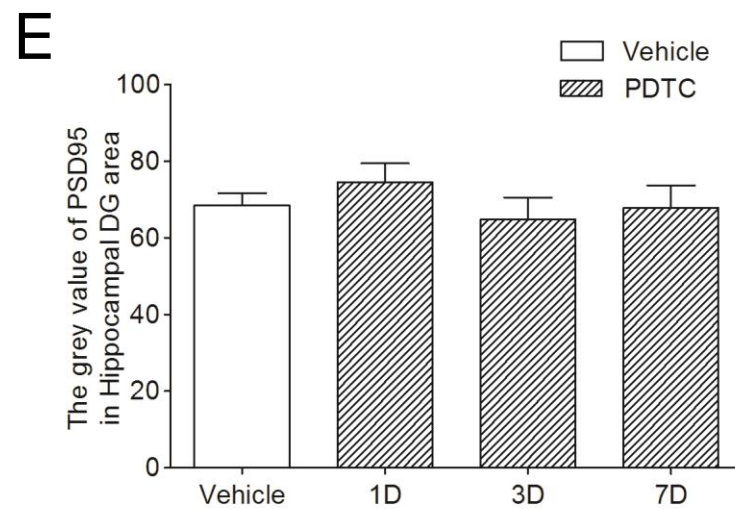

# Fig.3

## A

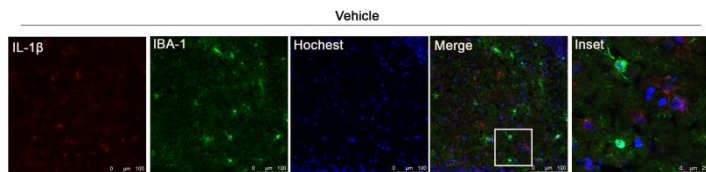

## B

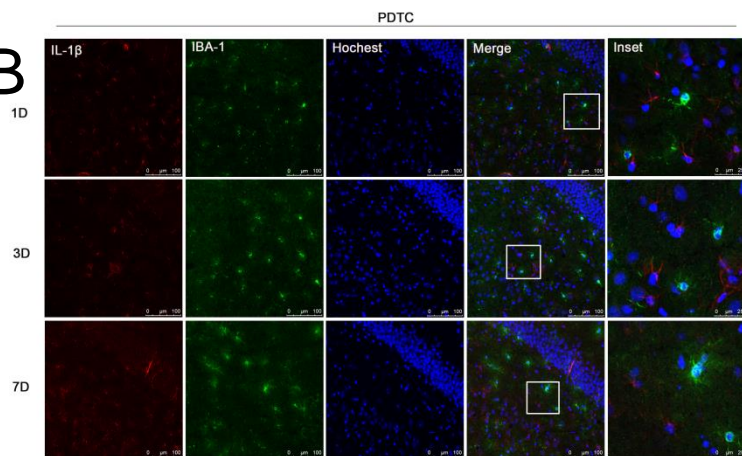

## C

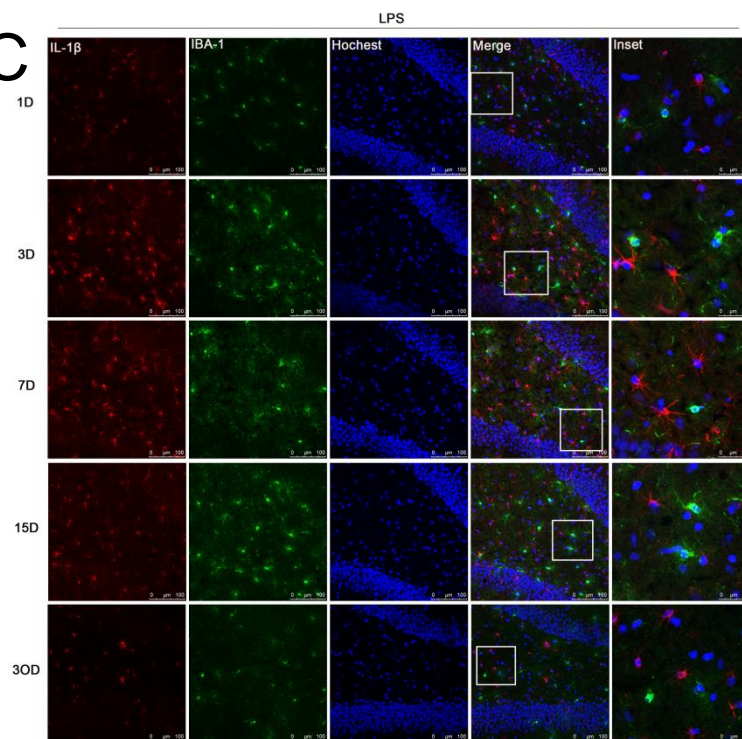

## D

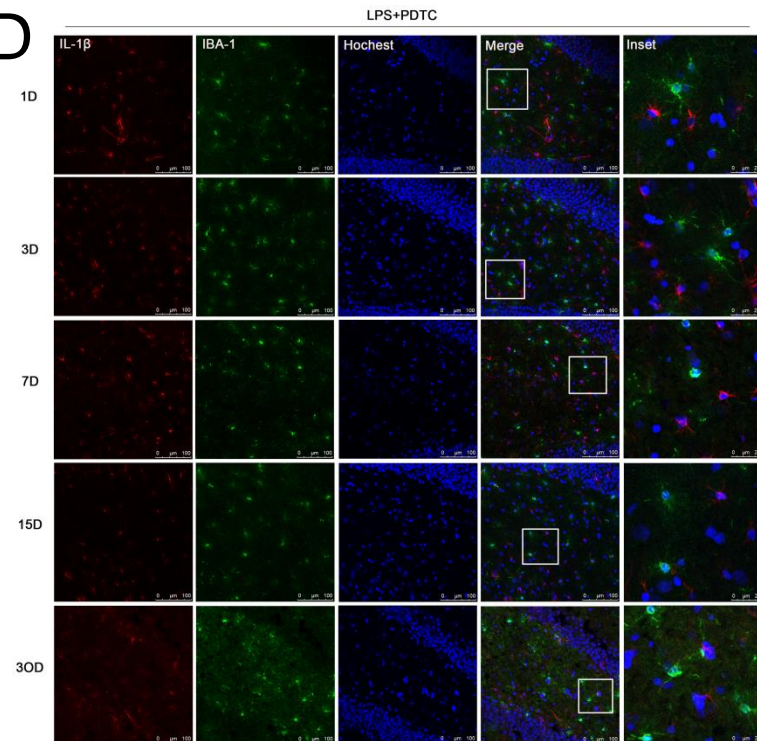

Fig.4

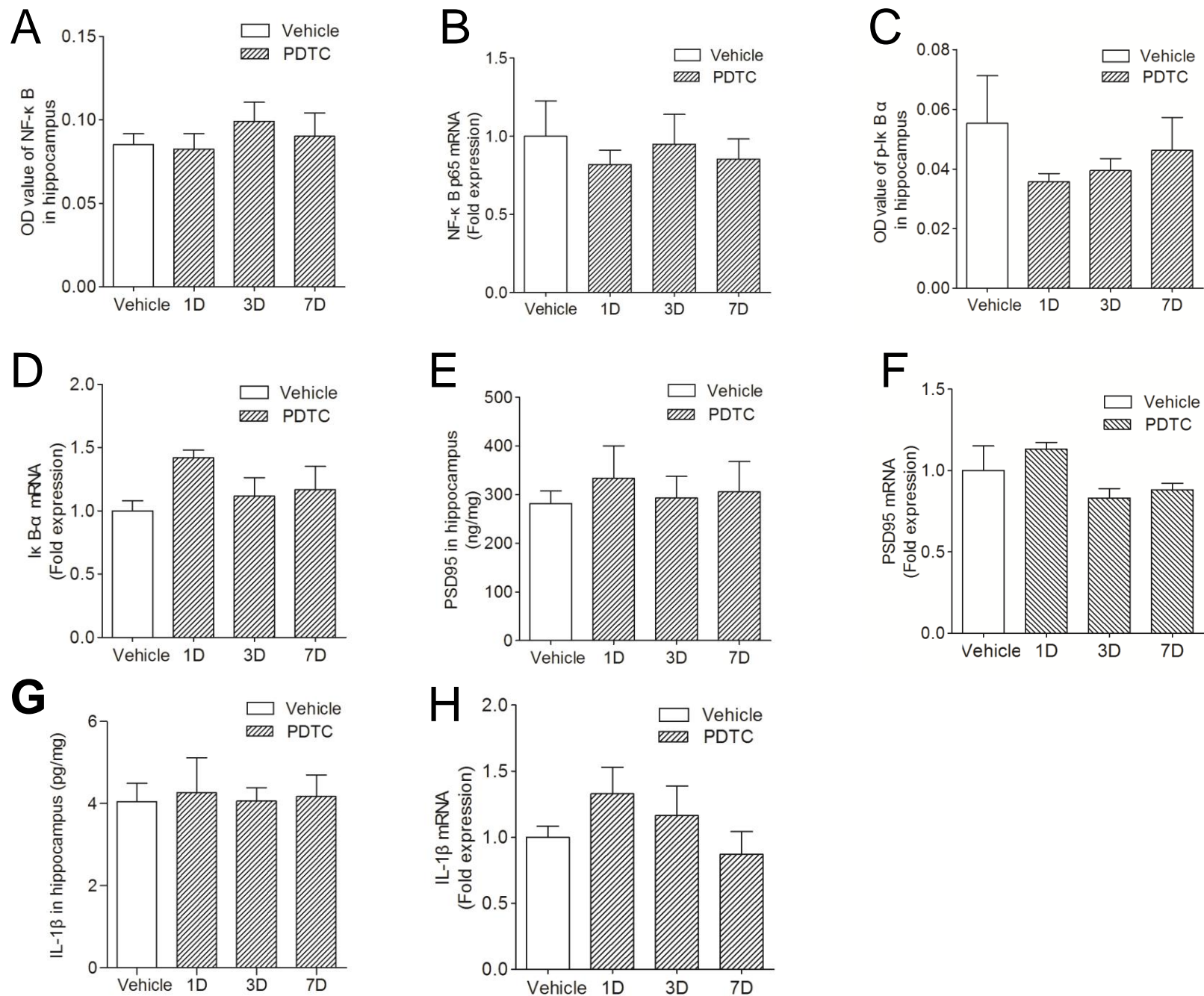

Supplement: Supplementary file 2 [file Presentation_2.pdf]
